# Pax6 modulates intra-retinal axon guidance and fasciculation of retinal ganglion cells during retinogenesis

**DOI:** 10.1038/s41598-020-72828-4

**Published:** 2020-09-30

**Authors:** Soundararajan Lalitha, Budhaditya Basu, Suresh Surya, Vadakkath Meera, Paul Ann Riya, Surendran Parvathy, Ani Venmanad Das, Krishnankutty Chandrika Sivakumar, Shijulal Nelson-Sathi, Jackson James

**Affiliations:** 1 Neuro Stem Cell Biology Laboratory, Neurobiology Division, Rajiv Gandhi Centre for Biotechnology, Thycaud PO, Thiruvananthapuram, Kerala 695 014 India; 2 Research Centre, The University of Kerala, Thiruvananthapuram, Kerala 695 014 India; 3 Regional Centre for Biotechnology, Faridabad, Haryana 121 001 India; 4 Cancer Biology Programs-12, Rajiv Gandhi Centre for Biotechnology, Thiruvananthapuram, Kerala 695 014 India; 5 Bioinformatics Facility, Rajiv Gandhi Centre for Biotechnology, Thiruvananthapuram, Kerala 695 014 India; 6 Interdisciplinary Biology, Rajiv Gandhi Centre for Biotechnology, Thiruvananthapuram, Kerala 695 014 India

**Keywords:** Visual system, Retina, Developmental biology, Differentiation, Neuroscience, Development of the nervous system

## Abstract

Intra-retinal axon guidance involves a coordinated expression of transcription factors, axon guidance genes, and secretory molecules within the retina. Pax6, the master regulator gene, has a spatio-temporal expression typically restricted till neurogenesis and fate-specification. However, our observation of persistent expression of Pax6 in mature RGCs led us to hypothesize that Pax6 could play a major role in axon guidance after fate specification. Here, we found significant alteration in intra-retinal axon guidance and fasciculation upon knocking out of Pax6 in E15.5 retina. Through unbiased transcriptome profiling between Pax6^fl/fl^ and Pax6^−/−^ retinas, we revealed the mechanistic insight of its role in axon guidance. Our results showed a significant increase in the expression of extracellular matrix molecules and decreased expression of retinal fate specification and neuron projection guidance molecules. Additionally, we found that EphB1 and Sema5B are directly regulated by Pax6 owing to the guidance defects and improper fasciculation of axons. We conclude that Pax6 expression post fate specification of RGCs is necessary for regulating the expression of axon guidance genes and most importantly for maintaining a conducive ECM through which the nascent axons get guided and fasciculate to reach the optic disc.

## Introduction

Retinal ganglion cells (RGCs) are the first-born neurons in the retina that carry the visual information from the retina to the brain visual centers through their long axons. During development, RGCs undergo three processes: axons from each RGCs grow and fasciculate with each other to reach the optic disc region, midline crossing at the optic chiasm region and finally reach the brain visual centers to make synaptic connections. Retinogenesis and retinal axon genesis are concurrent processes where in mice RGC generation begins at E10 and proceeds up to first post-natal week with a peak of RGC genesis at E14-E16^1^. The crucial function of axon guidance is a very tightly regulated process where, many transcription factors, a large family of axon guidance molecules such as ephrins, semaphorins, robos/Slits and extra-cellular matrix molecules (ECM) are involved in a spatio-temporal manner^2,3^. These molecules, mostly inhibitory in nature, act in concert to guide the axons to the optic disc where they bundle together to form the optic nerve. Although the expression of intra-retinal axon guidance molecules could be epigenetically regulated with precision as per the requirement, there is very little information available regarding the upstream regulators of the guidance genes. The most crucial stage for proper optic nerve formation is during intra-retinal fasciculation. During this stage, axons from each RGC emerges into the nerve fiber layer and fasciculate appropriately with the existing lead axons. It is essential to understand the early intra-retinal axon guidance and the genes involved in the process of development since, these conditions could be mimicked in the adult retina to facilitate proper fasciculation of the axons of transplanted RGCs and their bundling with existing nerves. We have previously observed robust integration of the transplanted cells into the host retina and minimal recovery of vision^4,5^. However, there is no evidence to show that the axons of the transplanted RGCs could properly fasciculate with the existing axonal bundles. Therefore, it is crucial to understand the regulatory mechanism by which nascent RGCs get fasciculated and bundled into the optic nerve. Since axon guidance is not regulated by a single gene but involves a coordinated regulation of multiple factors within the retina, we hypothesized that master regulator genes could govern this process.

Paired box transcription factor Pax6, one such master regulator which is evolutionarily conserved, plays a significant role in the development of eye and CNS by simultaneously regulating the expression of multiple gene sets^6^. Post mitotically, persistent Pax6 expression is observed in the retina and other brain regions^7^. We also observed an intense and persistent expression of Pax6 in RGCs and amacrine cells even after their genesis. This led us to hypothesize that Pax6 could have another vital role in intra-retinal axon guidance by regulating the expression of multiple factors that are essential for proper axon guidance and fasciculation. Several pieces of evidence support the notion that besides neurogenic potential, Pax6 can be involved in regulating the axon guidance function in the eye and also various regions of the brain^8–11^. However, none of these reports have shown how and which are the gene sets that Pax6 could be regulating to aid proper intra-retinal axon guidance and fasciculation of the nascent axons.

Here, we investigated the possible role of Pax6 in regulating the molecules involved in intra-retinal axon guidance using an in-vitro retinal explant culture and an in-vivo Pax6 conditional knockout mouse models. Our results showed a significant alteration in axon guidance and fasciculation upon knocking out of Pax6. To further identify the genes involved in axon guidance and fasciculation that are regulated by Pax6, we carried out RNA-Seq analysis in Pax6^−/−^ and Pax6^fl/fl^ (Control) retinas and found a significant up-regulation of ECM molecules in Pax6^−/−^ embryonic retinas. Recently, ECM molecules have gained significant recognition in regulating axon guidance in the neural system^12,13^. Our study thus assigns a new function to Pax6 in balancing the expression of ECM and classic axon guidance molecules in the embryonic retina, thereby facilitating proper axon guidance and fasciculation of the nascent axons of differentiating RGCs.

## Results

### Pax6 expression persists in RGCs during late retinogenesis to the adult stage

**Figure 1:**
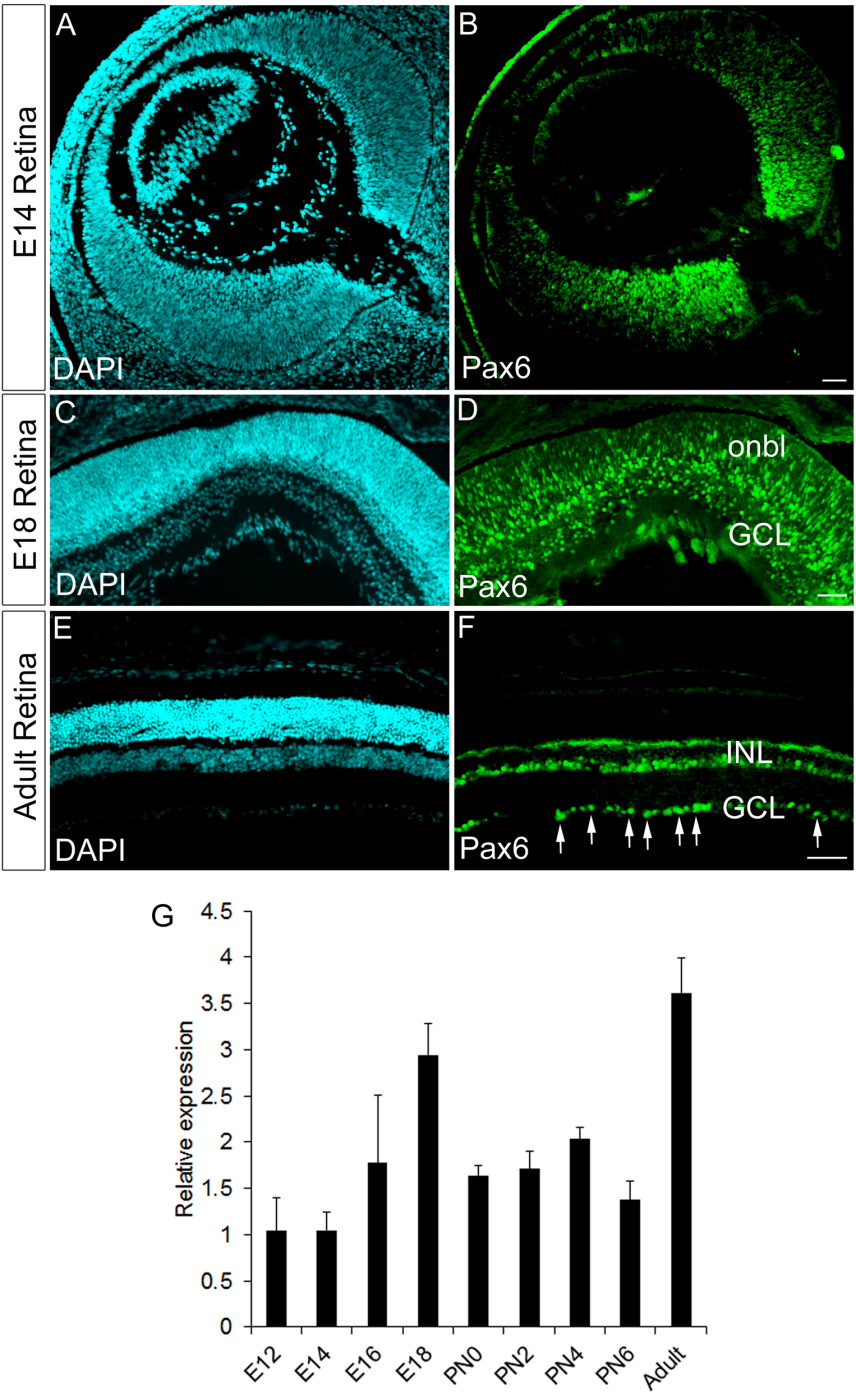
Persistent expression of Pax6 in differentiated RGCs. (**A**–**F**) Immunofluorescence analysis reveals expression of Pax6 in retinal progenitors, prospective RGCs and amacrine cells in E14 and of E18 retina (**B** & **D**) and persistent expression in the GCL of adult retina **(F)**. (**A**, **C** and **E**) Retinal sections were stained with DAPI. Scale bar—100 µm. (**G**) Spatio-temporal expression analysis with quantitative RT-PCR in the developing and adult retina shows a wave-like expression pattern for Pax6.

We checked the expression of Pax6 from an early retinal stage (E14, E18) and adult for its persistent expression after RGC fate specification (Fig. 1A–F). During E14 and E18, we have seen the expression of Pax6 in the progenitors, RGCs, amacrine cells and differentiating RGCs (Data not shown). However, in the case of an adult, continued expression of Pax6 is observed in the ganglionic cell layer (GCL), which is comprised of RGCs and amacrine cells (Fig. 1F) consistent with previous reports^7^. We also carried out temporal expression analysis of Pax6 from embryonic retina of E12 to the adult stage and have observed a wave-like pattern of Pax6 expression with a peak expression at E18 and in the adult (Fig. 1G). We already know that the early embryonic expression of Pax6 is vital for the fate specification of RGCs, but there is no reliable evidence regarding any function to indicate the retention of Pax6 expression beyond E18 extending to the adult stage^14^. Hence, we hypothesize that Pax6 could have a role in regulating intra-retinal axon guidance after fate specification in the retina.

### Pax6 modulates RGC axon extension and fasciculation

**Figure 2:**
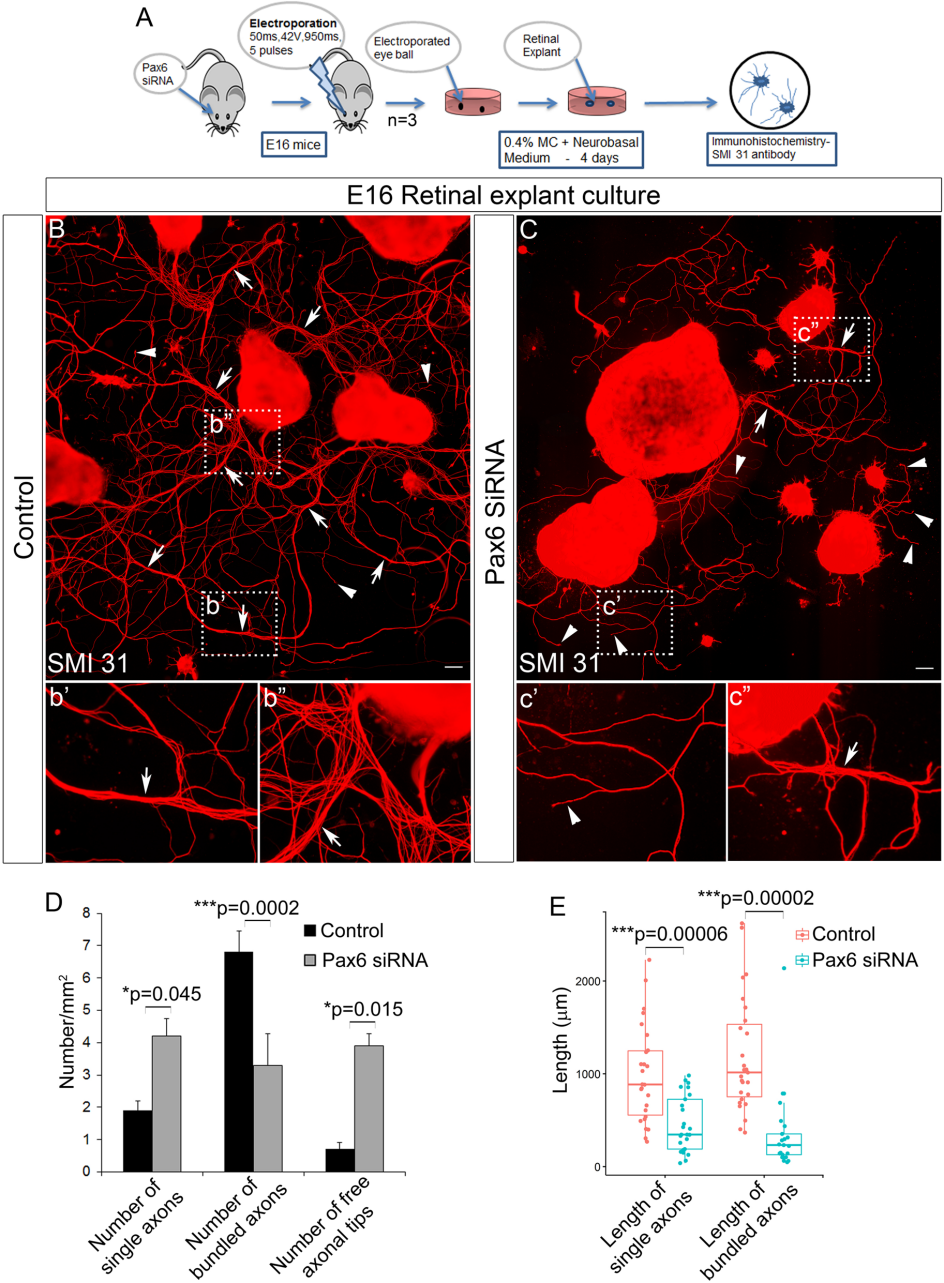
Perturbation of Pax6 in retinal explant cultures. (**A**) Schematic of the methodology adopted to perform perturbation of Pax6 in retinal explant cultures. (**B** and **C**) Ex-vivo retinal explant culture indicates a significant reduction in axonal outgrowth and fasciculation in Pax6 siRNA treated explants (**C**, **c**′, **c**″) compared to the control (**B**, **b**′, **b**″). Scale bar—100 µm, **B** and **C** represent images generated by stitching multiple images using Photoshop. (**D** and **E**) Quantification of the neurite numbers (D; p < 0.05) and the length of the axons/bundles (**E**; t-test; p < 0.001) for the explant culture represented in **B** and **C** using ImageJ software. n = 3, 3 independent explant culture preparation were made. Error bars indicate SEM from three biological replicates.

Initially, to understand the functional role of Pax6 post fate specification, we down-regulated Pax6 at E16 with siRNA, maintained retinal explant cultures for 4 days and the axons were visualized with SMI31 antibody (Fig. 2A). In an explant culture of the retina, only the RGCs will be able to generate long outgrowing axons. Our results showed a drastic difference in the axon outgrowth, extension, and fasciculation in Pax6 siRNA electroporated retina compared to the control (Fig. 2B–C). Further analysis revealed a significant increase in the number of single axons (n = 3; p = 0.045) and free axonal tips (n = 3; p = 0.0159) in Pax6 down-regulated retina. There was also a significant reduction in the number of bundled axons (n = 3; p = 0.0002), length of the bundled axons (n = 3; t-test; p = 0.000025) and length of single axons (n = 3; t-test; p = 0.000061) in these siRNA treated explants (Fig. 2D–E). These observations indicated that down-regulation of Pax6 in RGCs could lead to diminished axonal outgrowth which in turn made it hard for axonal tips to fasciculate thereby resulting in an increased number of free axonal tips and single axons (Fig. 2b′–b″ and c′–c″). These results suggested another significant role for Pax6 in RGCs other than fate specification. These are in line with the previous reports where axons are unable to reach their targets when Pax6 is knocked out^8,11^. Although we observed a fasciculation defect in Pax6 down-regulated retinas, we cannot completely rule out improper RGC maturation that can lead to reduced axonal outgrowth.

### Knocking out of Pax6 after RGC fate specification showed altered fasciculation

**Figure 3:**
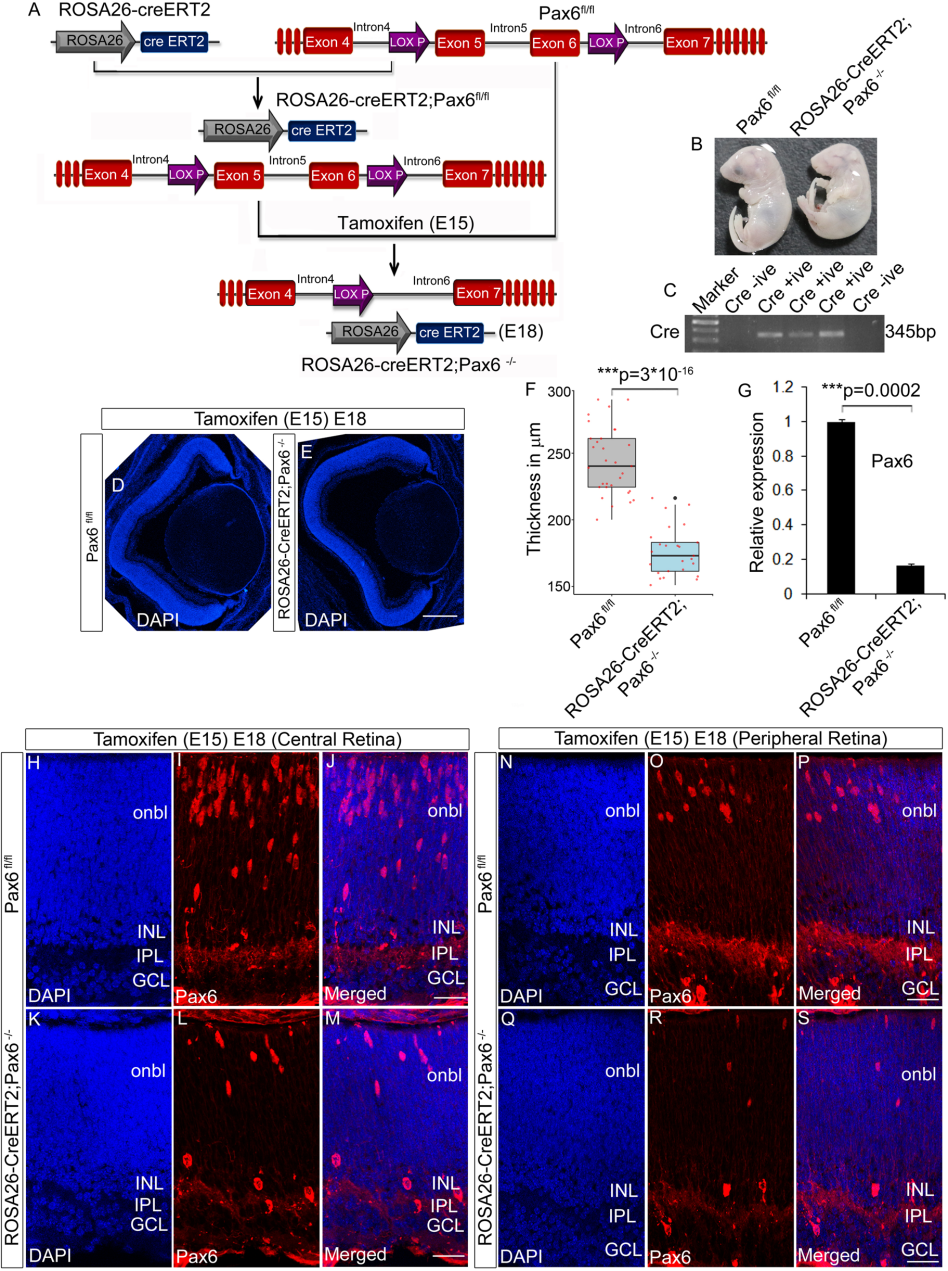
Generation of Pax6 cKO (Pax6^−/−^) embryos. (**A**) Schematic of the breeding strategy used in the study to generate Pax6^−/−^ embryos after tamoxifen injection. (**B**) Pax6^fl/fl^ and Pax6^−/−^ embryos collected at E18 after tamoxifen injection at E15 display marked phenotypic differences. (**C**) Genotyping PCR to screen Pax6^fl/fl^ and Pax6^−/−^ animals based on the presence of cre. (**D**–**F**) DAPI staining of Pax6^fl/fl^ and Pax6^−/−^ embryos revealed a reduction in the thickness of the neuroretina of Pax6^−/−^ embryos (**E**) compared to the Pax6^fl/fl^ (**D**) embryos. Thickness of the retina is plotted as box plot using R program (**F**; t-test; p < 0.001). Scale bar—25 µm (**G**) Quantitative expression analysis for *Pax6* in Pax6^fl/fl^ and Pax6^−/−^ retina showed a significant reduction in the expression level (p < 0.001). (**H**–**S**) Immunostaining with N-terminal Pax6 antibody showed a significant reduction in the number of Pax6 positive cells in both the central (**I**, **L**) and peripheral region (**O**, **R**) of Pax6^−/−^ retina compared to Pax6^fl/fl^ retina. As such, the numbers of Pax6 positive cells are lesser in the peripheral retina (**O**) compared to the central retina (**I**). Scale bar—25 µm. n = 3, at least 3 embryos each of Pax6^fl/fl^ /Pax6^−/−^ from 3 pregnant animals were collected. Error bars indicate SEM from three biological replicates.

Since RGC genesis and axon extension are concurrently regulated processes (from, E10-E18)^1^, it is challenging to study the post fate specification function of Pax6 separately. To overcome this, we knocked out Pax6 at the peak of RGC genesis (E15.5) by administering Tamoxifen (Fig. 3A) and confirmed the Pax6^−/−^ embryos by checking the presence of Cre through genotyping (Fig. 3C). Knocking out of Pax6 at E15.5 would give rise to two possible scenarios. First, retina would have a residing fraction of RGCs that were born earlier than E15 and have already extended their axons to the optic disc. Second, there would be newly born RGCs consisting of both the nascent mature RGCs that are starting to sprout their axons and find their way to the retinal optic disc as well as immature RGCs that are yet to sprout their axons. Additionally, there will also be a reduction in new RGC genesis after Pax6 knockout leading to a reduction in late-born RGCs (Fig. S2B, F). Both the mature and immature RGCs will be found more in the central retina compared to the peripheral retina since RGC genesis begins from the central retina surrounding the optic disc and spreads out to the peripheral retina^15^. Morphologically Pax6^−/−^ embryos appeared smaller with small head than the controls but without a marked difference in the size of the eye (Fig. 3B). Confirmation of Pax6 KO embryos was carried out with qRT-PCR, which showed a significant reduction (n = 3; p = 0.000251) in Pax6 expression compared to Pax6^fl/fl^ retinas (Fig. 3G). We also analyzed the morphology of the retina that showed significant thinning (n = 3; t-test; p = 3 × 10^–16^) in the cross-section of the eye compared to the Pax6^fl/fl^ (Fig. 3D–F). Confirmation with Pax6 antibody that recognizes N-terminal of the protein revealed a drastic reduction of Pax6 positive cells in the RGC layer as well as outer neuroblastic layer (Onbl) both in the central (Fig. 3H–M) and peripheral retina (Fig. 3N–S). This observation suggested that few of the fate specifying genes in the early retinogenesis could also have been affected by the loss of Pax6. In order to check this out, we carried out immunofluorescence analysis using specific antibodies for other retinal cell types. Analysis with Ap2α, recoverin, and calbindin antibody showed a significant reduction in amacrine cells (Fig. S1B,F; n = 3; p = 0.028), photoreceptors (Fig. S1J,L; n = 3; p = 0.046) and horizontal cells (Fig. S1P; n = 3; p = 0.021) respectively in the Pax6^−/−^ retina compared to the Pax6^fl/fl^.

We next examined the number of RGCs that are born after Pax6 was knocked out. To address that we have used Brn3 antibody that marks all types of RGCs and the results indicated a significant reduction in the percentage of RGCs in Pax6^−/−^ embryos (Fig. S2B and I; n = 3; p = 0.046), these observations indicated that Pax6 has a significant role in the fate specification of retinal subtypes and also substantiating the reduction of retinal thickness. To rule out the possibility of cell death mediated reduction in the number of cells in the Pax6^−/−^ retina, we performed TUNEL assay in both Pax6^fl/fl^ and Pax6^−/−^ retinal sections but did not observe any significant difference in the number of cells that underwent apoptosis in Pax6^−/−^ retina, (Data not shown) indicating altered retinal differentiation than cell death.

**Figure 4:**
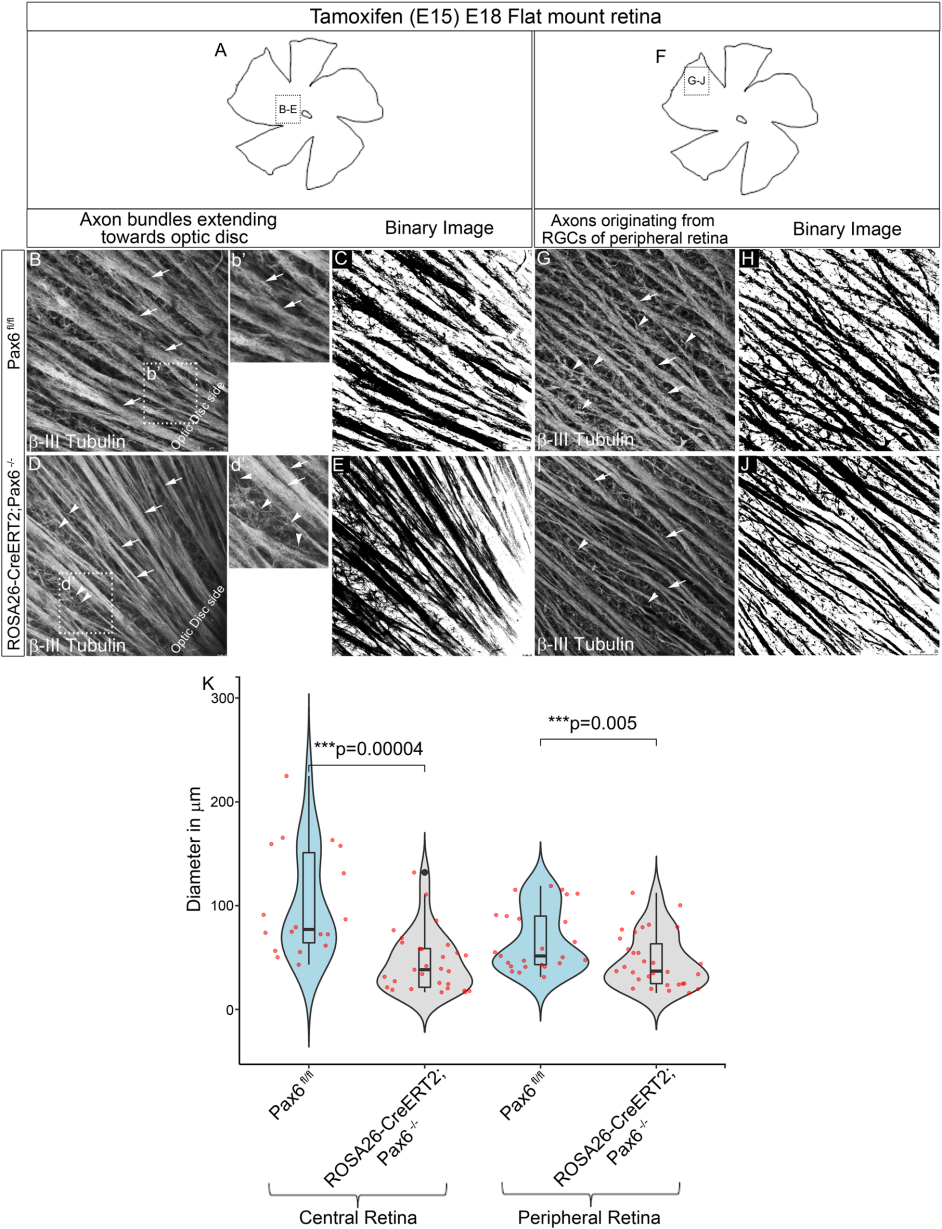
Pax6^−/−^ retina showed altered intra-retinal axon guidance and fasciculation. (**A**–**J**) β-III Tubulin stained flat mount retinas showing the central region near optic disc (**A**) and peripheral region (**F**) indicating a significant reduction in thickness of the bundles along with many defasciculated axons in Pax6^−/−^ retina (**D**, **d**′ and **I**) compared to the Pax6^fl/fl^ retina (**B**, **b**′, **G**). Binary images of the same were created using ImageJ software (**C**, **E**, **E** and **J**). Scale bar—25 µm. (**K**) Violin plots depicting thickness of the bundles in both central and peripheral region measured using Neuromantic software indicating that there is a significant reduction in the thickness of bundles in both central and peripheral region of the Pax6^−/−^ retina. n = 5, 5 retinal flat mounts of embryonic retina (Pax6^fl/fl^ and Pax6^−/−^) were stained from 5 independent pregnant mothers. Error bars indicate SEM from five biological replicates (Wilcoxon test; p < 0.001).

To further investigate the role of Pax6 in intra-retinal axon guidance, retinal flat mounts stained with β-III tubulin were analyzed for axon bundle thickness/width in control and Pax6^−/−^ retinal flat mounts in both central and the peripheral retina using Neuromantic V1.6.3. In central region (Fig. 4A) of Pax6^−/−^ retina we observed that most of the axons were not fasciculated with the existing axon bundles as indicated by arrowheads compared to the control (Fig. 4B-b′, D-d′′) and are easily visualized in the binary image (Fig. 4C,E). The aberrant axons were found to be predominantly present in the central retina compared to the peripheral retina because more RGCs are found in the central retina than in the peripheral retina (Fig. 4E,J). Looking at the gross morphology, we could also find a significant reduction in thickness of axon bundles in the central region (n = 5; Wilcoxon test; p = 0.0004) of the retina near the optic disc region in the Pax6^−/−^ compared to the control (Fig. 4B,D,K). We also found a contrasting significant decrease in defasiculated axons in peripheral Pax6^−/−^ retina (Fig. 4F) compared to control as indicated by arrowheads (n = 5; p = 0.005, Fig. 4G–J,K). The RGCs that express the aberrant axons could be the nascent matured RGCs that had just started sprouting their axons. We observed ~ 75% reduction in the number of RGCs (Fig. S2) but, it is still not clear whether the newly formed RGC axons have lost their ability to fasciculate together with the existing axon bundles or they are straying away from them. The observed significant reduction in the axon outgrowth and fasciculation may not be exclusively due to axon misguidance but can also be due to failure of RGC maturation. Reduction in the thickness of axon bundles could also be due to the reduction in the number of RGCs in the Pax6^−/−^ retina (Fig. S2I, n = 3; p = 0.046) as well as the failure of the RGCs to mature properly to extend their axons (Fig. S2F). Although we could not differentiate between these possibilities, we speculate that axon-axon interactions might be lost in Pax6^−/−^ retinas compared to controls. Altogether, these findings indicate that Pax6 is indispensable for regulating the intra-retinal axon guidance and fasciculation.

### RNA-Seq reveals down-regulation of retinal development gene sets in Pax6^−/−^ retinas

**Figure 5:**
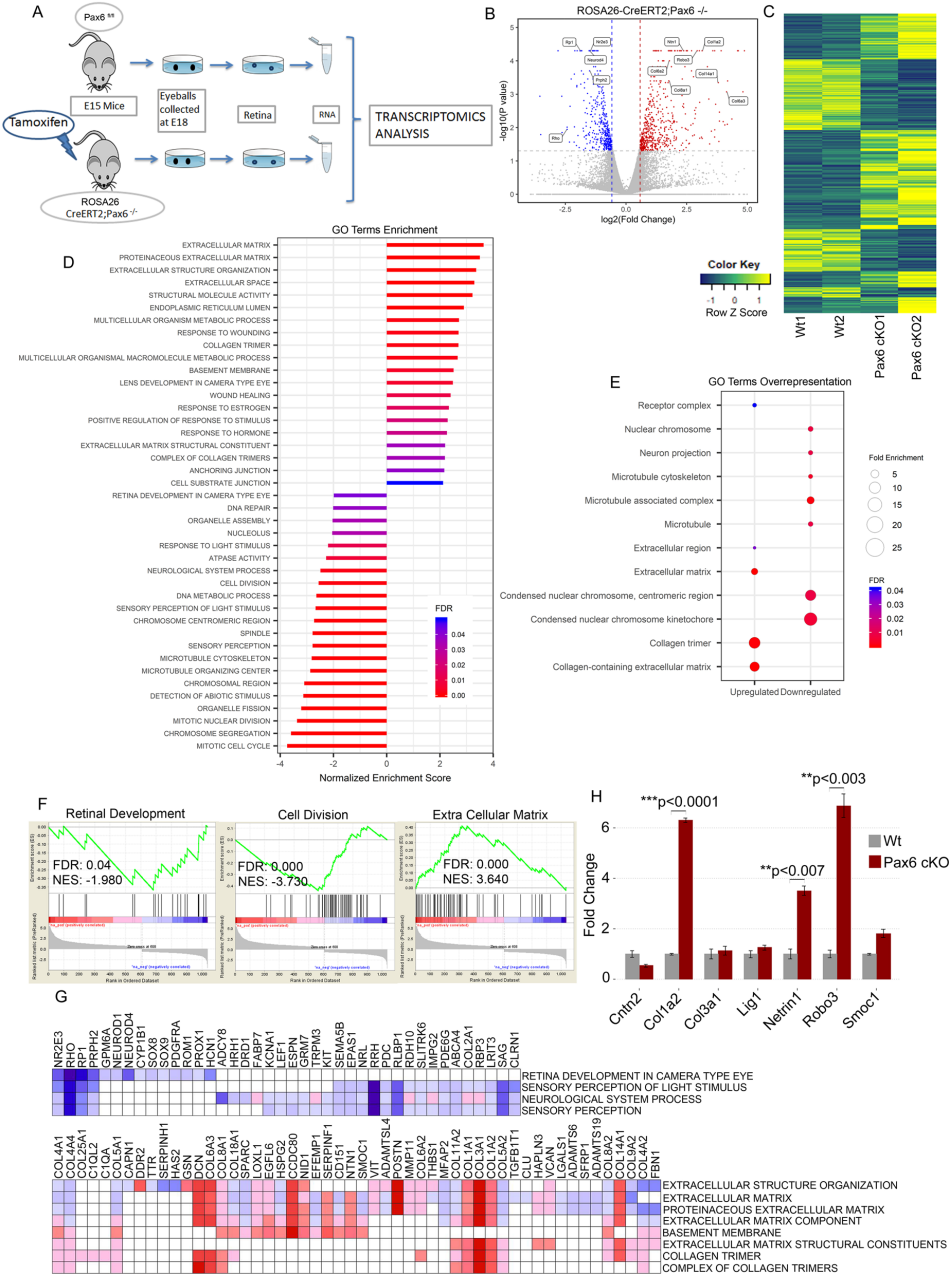
RNA-Seq data reveals altered expression of ECM gene sets. (**A**) Schematic for the protocol employed to carry out RNA-Seq analysis. (**B**) Differential expression analysis shows changes in gene expression in Pax6^fl/fl^ vs Pax6^−/−^ retina. The data for all genes is plotted as log_2_ fold change versus the − log_10_ of p-value. The threshold values are shown in dashed line (p < 0.05, Fold change > 1.5). Red dots and blue dots indicate significantly up-regulated and down-regulated genes, respectively. Few genes related to the ECM and retinal development is labeled with gene symbol. (**C**) Heat-map shows fold change of differentially expressed genes in duplicate samples of Pax6^fl/fl^ (Wt-Control) and Pax6^−/−^ (cKO) retina. Values are normalized as row Z scores. (**D**) GO analysis by statistical enrichment test performed using java GSEA 3.0. (**E**) GO analysis by statistical overrepresentation test (Cellular Component). (**F**) Gene Set Enrichment plots from GSEA of Pax6^fl/fl^ vs Pax6^−/−^ (NES- Normalized enrichment score; FDR- False discovery rate). (**G**) Leading edge analysis of enriched ECM and retina related gene sets shows no overlap between these two gene sets. The column represents gene that constitutes the primary enrichment in different gene sets, which is represented in a row. Expression values are represented as range of colours (red, pink, light blue and dark blue) show a range of expression values (high, moderate, low, and lowest) respectively. (**H**) Axonal guidance genes and collagen subunits that were differentially expressed were confirmed with qRT- PCR analysis. Error bars indicate SEM from three biological replicates.

To identify the functional molecules, deregulated genes and signaling pathways that are associated with the loss of Pax6, we have analyzed the transcriptomic signatures of both Pax6^fl/fl^ and Pax6^−/−^ retinal tissue (Fig. 5A). We performed an unbiased deep RNA sequencing and found that 1055 genes were differentially expressed (p < 0.05, log2FC > 0.58, Fig. 5B). With this threshold, technical replicates produced consistent results, the comparison showed that 627 genes were up-regulated and 428 genes were down-regulated in Pax6^−/−^ retina indicating the molecular control of master regulator protein Pax6 (Fig. 5C). Next, we performed Gene Ontology (GO) analysis of same Differentially Expressed Genes (DEG) by two different statistical methods: Statistical Enrichment test: Gene Set Enrichment Analysis (GSEA, Fig. 5D,F) and Statistical Over-representation test (Fig. 5E). Both statistical methods gave us the confidence that ECM, basement membrane and collagen molecules were significantly overexpressed (Fig. 5D–E), whereas cell division, retinal development (Fig. 5D), neuron projection (Fig. 5E) related genes were down-regulated. These were further confirmed with qRT-PCR analysis (Fig. 5H).

The down-regulated gene sets included genes such as NeuroD1, NeuroD4, Nr2e3, Rp1, Prph2, and Rho that are involved in the fate specification of other retinal neurons (Fig. 5G). These gene sets are already known to be critical for the development and fate specification of the retina^16–21^. Consistent with our previous results, the reduction of amacrine cells (Fig. S1B,F ) could attribute to the significant decrease in NeuroD in the Pax6^−/−^ retina. There was also a drastic reduction in Sox8 and Sox9 in the Pax6^−/−^ retina. Sox8 and Sox9 are expressed in the proliferating progenitors and later needed for the specification of Müller glial cells^22^. Interestingly, we could observe down-regulation of Gpm6a in the Pax6^−/−^ retina, which plays an essential role in transducing the signals from ECM as neurons express them^23^. This could be one of the reasons that the Pax6^−/−^ retinal axons are not receiving or become unresponsive to the signals from the ECM as improper neurite extension is observed. Another significantly down-regulated group of genes directly involved in cell division was also observed (Fig. 5F).

### Loss of Pax6 modulates the expression of genes coding for retinal extracellular matrix molecules

We found up-regulation of ECM molecules (Fig. 5F–G) in Pax6^−/−^ retina and were interesting due to the observed loss of axon fasciculation (Fig. 4A–J). ECM forms an essential component of basement membrane consisting of collagen, laminin, fibronectin, etc., onto which the retinal axons are fasciculated and guided towards the optic nerve head during retinal development. Out of all the ECM genes that get up-regulated, collagens are of interest since they are essential for neurite outgrowth and axon guiding to their final destinations (Fig. 5G). Our RNA-Seq data showed a significant up-regulation of Col1a1, Col1a2 and Col3a1 molecules, which are the fibrillar form of collagen that has substantial roles in the neuroblast migration^12^. In addition to the collagens, other ECM proteins such as Smoc1, Mmp11, Postn, Has2, Hspg2 and Nid1 are also up-regulated in the retina of Pax6^−/−^ embryos. This severe up-regulation of ECM molecules may not be supportive for the axon to move and fasciculate with the existing axonal bundles. Therefore, Pax6 might affect the formation and distribution of ECM. That raises a possibility that some of the ECM would be secreted by the RGCs as well as other Pax6 expressing cells in the retina, which needs to be further investigated.

Although the up-regulation of many collagen molecules was confirmed through RNA-Seq analysis, how they form structural assemblies is a plausible question. Collagen structures can create polymerization dynamics^24^ that rules for the structural assembly of their domains, leading to intriguing new insights into collagen assembly behavior. Moreover, we assume that the formation of abnormal assemblies of identical collagen proteins in the ECM may interfere with each other by some manner and conceal the interacting molecules from the axonal receptors or the axons which result in losing their contact points on the basement membrane. Whether this increased collagen expression can misguide the axons is not yet known. From this point of view, it is essential to understand how the polymerization dynamics of collagen matrix gets altered when they are over-expressed. To efficiently explore the collagen interaction dynamics, we used SymmDock server^25^ for symmetrical protein–protein docking. Several models of different conformers of collagen Col1a2 (PDB:5CTD, B chain) were successfully matched in the order of their structural symmetry, and partial structures were fitted into larger assemblies. Fig. S3 summarizes various symmetric conformations of collagen Col1a2 sorted according to their atomic contact energies (ACE, Fig. S3J) and 3D transformation. It is interesting to note that as the symmetry increases in a specific order (4mer, 5mer, 8mer, 9mer, and 10mer), the dynamics and stability of identical collagen polymers are greatly intensified. Therefore, the similarity in the overall structure of identical collagen could lead to higher-order symmetric conformers that induce abnormal assemblies and transformation in ECM thus creating a hostile environment for proper growth and fasciculation of the axons (Fig. S3).

### Pax6 modulates ECM composition for proper fasciculation of axons

**Figure 6:**
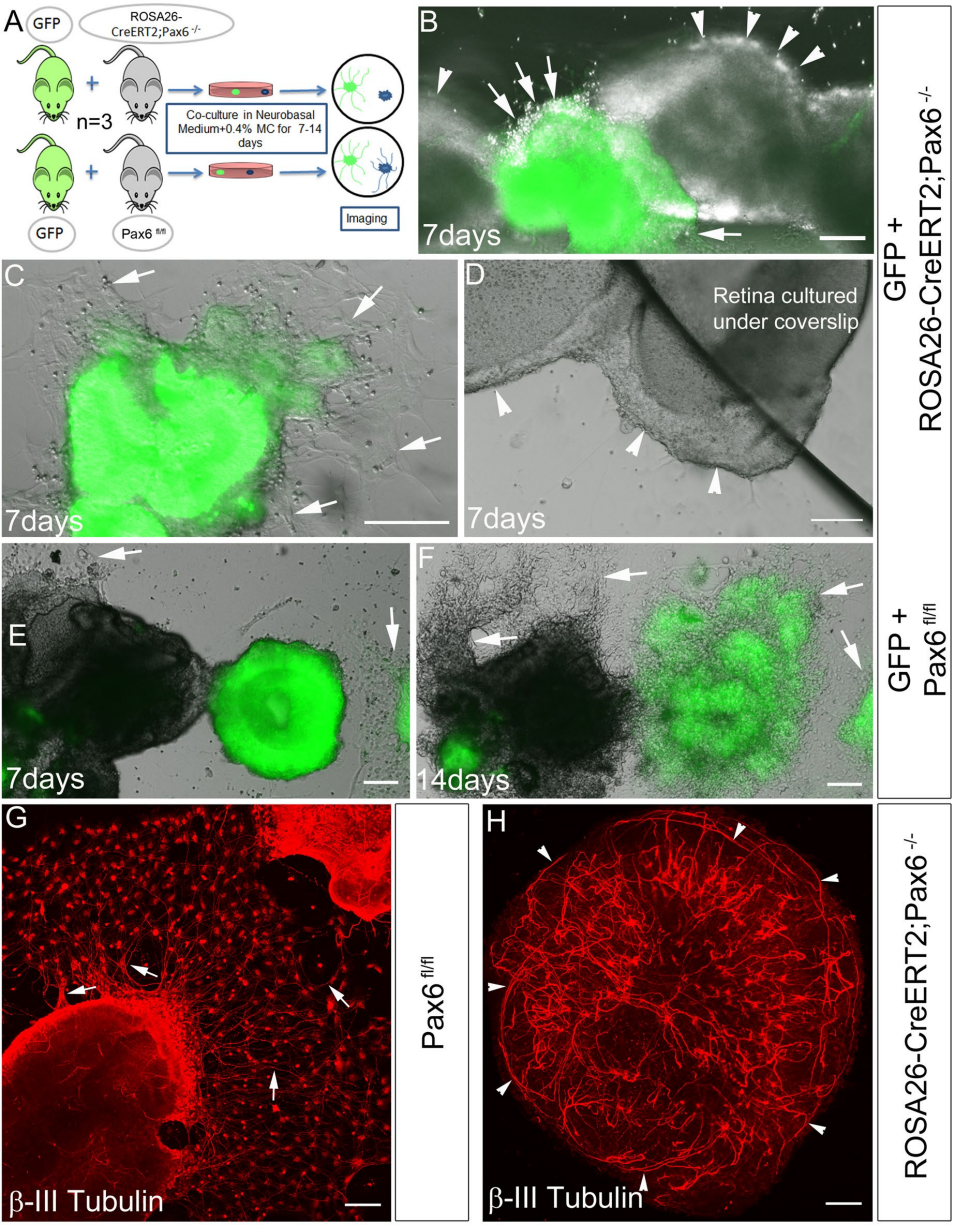
Pax6 controls the proper attachment and neurite outgrowth in the retinal explants. (**A**) Schematic of the methodology adopted to perform this experiment. (**B**–**F**) Co-culturing retinal explants from control GFP and Pax6^−/−^ embryos (**B**–**D**) Pax6^−/−^ retinas displayed poor attachment to the culture dish (arrowheads **B** and **D**). (**D**) Pax6^−/−^ retinal tissue was forced down with coverslip but still lacked attachment potential. (**E** and **F**) GFP and Pax6^fl/fl^ embryos co-cultured together showed robust axon and neurite outgrowth displaying proper attachment (Arrows, **B** & **C**, **E** & **F**). Scale bar—100 µm. (**G** and **H**) β-III tubulin stained retinal explants from Pax6^fl/fl^ and Pax6^−/−^ retina, showed altered axonal outgrowth and fasciculation in Pax6^−/−^ retinas. n = 3, 3 independent explant culture preparations were made. Scale bar—100 µm.

From our data, it is indicative that Pax6 could modify ECM composition facilitating axon guidance/fasciculation. Previously, we observed with explant cultures that there was no proper attachment of Pax6^−/−^ retinas onto the culture dishes (Data not shown). Taken together, it appears that altered ECM could be the cause for the altered axon outgrowth leading to non-attachment of retinal explants. To prove this, we co-cultured retinal explants from normal E18 GFP expressing embryos along with Pax6^fl/fl^ and Pax6^−/−^ retinal explants for 14 days (Fig. 6A, see methods). Both Pax6^fl/fl^ and Pax6^−/−^ retinas were obtained from littermates. By 7^th^ day, the co-cultured explants from the normal GFP and Pax6^fl/fl^ retina started attaching to the culture dish with visible neurites. However, other wells having GFP and Pax6^−/−^ co-cultured explants, only GFP explants were attached whereas Pax6^−/−^ explants did not show any attachment (Fig. 6B,C,E). Since the Pax6^−/−^ retinal explants were continually floating away, we artificially forced them onto the culture dish by placing coverslips over them but did not observe any attachment or axonal outgrowth ex-vivo, indicating alteration in the composition of secreted ECM (Fig. 6D). Whereas by 14 day, GFP and Pax6^fl/fl^ explants attached to the culture dish with profuse extended axon network (Fig. 6F) but not Pax6^−/−^ retinal explants. We also stained 7 day cultured Pax6^fl/fl^ and Pax6^−/−^ retinal explants with β-III tubulin antibody to check if at all the axons are formed in the Pax6^−/−^ retinal explants. We observed attachment and axon outgrowth/fasciculation in Pax6^fl/fl^ explants (Fig. 6G) whereas, Pax6^−/−^ explants did not attach and the axons were found to be growing within the explant itself (Fig. 6H). The possible reason of non-attachment could be attributed to the surface charge redistribution in deposited collagens of ECM in Pax6^−/−^ retina that could have prevented outgrowth of axons and fasciculation. These results indeed prove that ECM alteration could lead to the defects in the attachment of explants, and neurite outgrowth ex-vivo, similar to the altered fasciculation of axons observed in-vivo in Pax6^−/−^ retinas.

### Pax6 regulates axon guidance genes EphB1 and Sema5b

**Figure 7:**
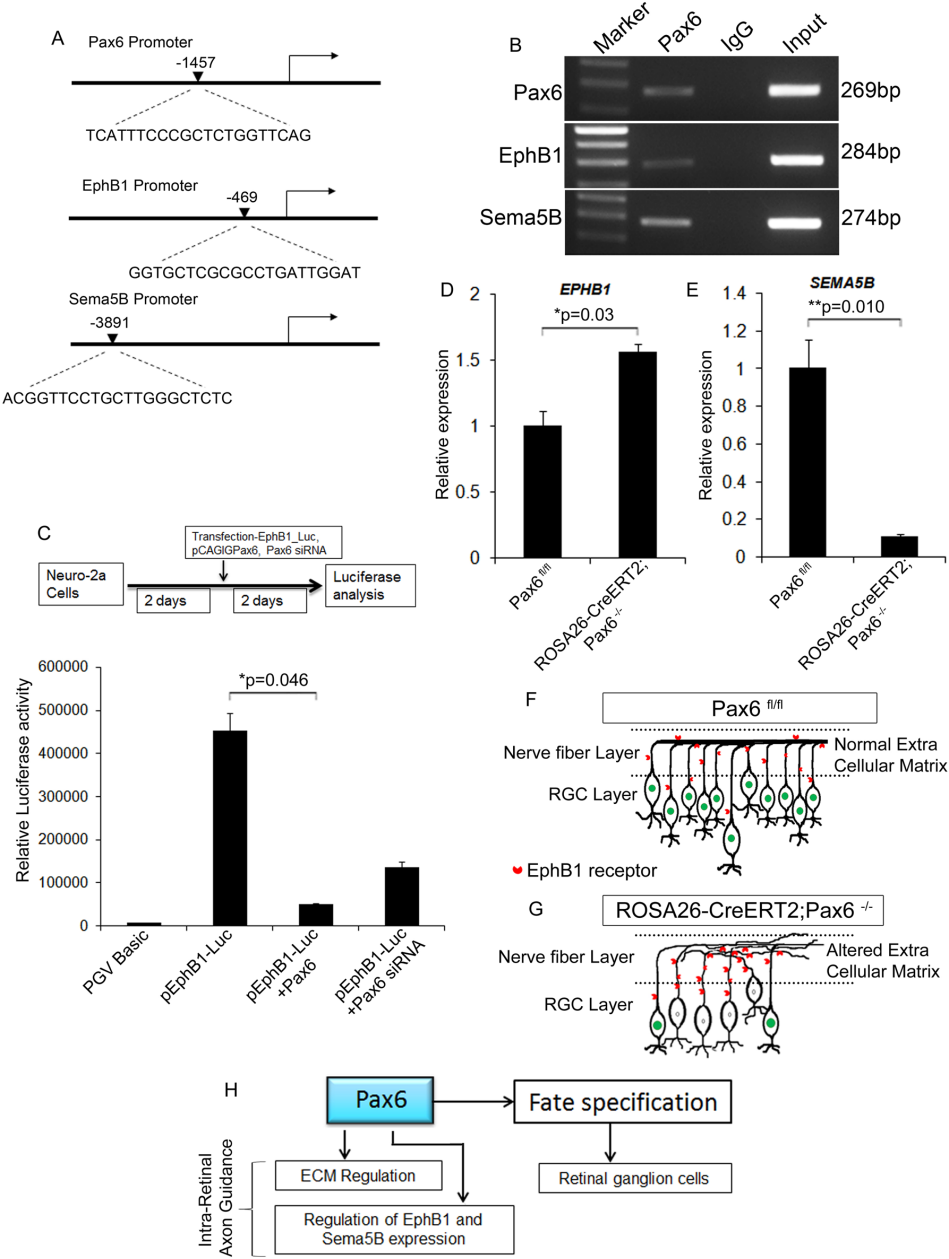
Pax6 directly regulates axon guidance genes. (**A**) In silico analysis of the promoters of Pax6, Ephb1 and Sema5b showing Pax6 binding sites. (**B**) ChIP-PCR on E18 retina showed that Pax6 binds to the promoters of Pax6, Ephb1 and Sema5b. (**C**) Luciferase analysis showed negative regulation of Ephb1 by Pax6. (**D** and **E**) qRT-PCR study showed a significant up-regulation of EphB1 expression (p < 0.05) in the Pax6^−/−^ retina compared to Pax6^fl/fl^ whereas, expression of Sema5b was significantly reduced (p < 0.001). (**F** and **G**) Schematic showing the intra-retinal axon fasciculation defects in the Pax6^−/−^ (**G**) retina compared to Pax6^fl/fl^ (**F**), that is due to the alteration in the ECM composition. (**H**) Schematic depicting the novel functions of Pax6 in the retina by regulating the expression of ECM and axon guidance genes. n = 3, 3 independent cell culture and transfection experiment were carried out in triplicate wells.

Although we could observe a severe up-regulation of ECM molecules, we extended our analysis with a few essential axon guidance genes that showed differential expression levels in the absence of Pax6. These genes were probed in-silico for Pax6 binding sites on their promoters and found direct binding sites for Pax6 on the promoters of Pax6, Sema5b, and EphB1 (Fig. 7A). To confirm binding ChIP-PCR was carried out on E18 retina with Pax6 antibody and confirmed that Pax6 can directly bind to the promoters of EphB1 and Sema5B (Fig. 7B). Further interactions, were confirmed with luciferase analysis using EphB1 promoter constructs and over-expressing and down-regulating Pax6 in N-2a cells that endogenously express Pax6. Significant reduction (n = 3; p = 0.046) and increase of luciferase activity was observed upon over-expression and down-regulation of Pax6 respectively (Fig. 7C). These results indicated that Pax6 has a negative regulation on EphB1 upon binding to its promoter (Fig. 7C). To also understand the regulation of EphB1 and Sema5B by Pax6 in-vivo, we carried out gene expression analysis between the Pax6^fl/fl^ and Pax6^−/−^ retinal tissues. We could see an increase in the expression of EphB1 in the Pax6^−/−^ retina (n = 3; p = 0.03; Fig. 7D) and the expression of Sema5B was significantly reduced (n = 3; p = 0.01) in the Pax6^−/−^ retina when compared to the control (Fig. 7E). Therefore, from our results, it is clear that Pax6 negatively regulates EphB1 in-vivo, thereby it could be influencing intra-retinal axon guidance.

Based on our results here, we have shown a new role for Pax6 in addition to RGC genesis. Various reports and our data have shown that knocking out of Pax6 during RGC genesis drastically reduces the number of differentiated RGCs (Fig. S2) as well as RGC axons might lose their way to the optic disc (Fig. 7F–G). Further, during the maturation phase of RGC, Pax6 plays a critical role in the formation of a conducive ECM and regulating expression of axon guidance genes thus properly maintaining intra-retinal axon guidance and fasciculation (Fig. 7H).

## Discussion

Various studies have demonstrated the pleiotropic role of Pax6 in the neural development where it plays a significant role in cell cycle, fate specification, neuronal differentiation, migration, cell adhesion and axon guidance^6^. Pax6 is considered as the master regulator of eye development since eye morphogenesis is arrested in the Pax6^−/−^ mice^26^. Pax6 mediates all these functions by regulating various downstream molecules in a context-dependent manner. Although several studies have pointed out the importance of Pax6 in retinal differentiation, the role of Pax6 in stage-specific regulation of retinogenesis and intra-retinal axon guidance and how it is mediating the functions are mostly unexplored.

In the retina, different cells are formed in an overlapping manner with the activation of a specific set of genes during embryonic and post-natal stages^27^. Our results showed that when Pax6 is eliminated in all retinal cells from E15.5, the number of RGCs, amacrine cells, horizontal cells and photoreceptors are drastically decreased in the Pax6 mutant mice compared to the control. Further looking into the genes that are altered between Pax6^fl/fl^ and Pax6^−/−^ retina, we have analyzed our RNA-Seq data and found that many of the genes that are essential for retinal development are severely affected by the loss of Pax6. The most important genes are NeuroD1, NeuroD4, Nr2e3, Rp1, Prph2, and Rho, which are involved in the fate specification of retinal neurons. These genes showed a drastic reduction in their expression level in the absence of Pax6. The reduction in the number of RGCs, amacrine cells, horizontal cells and the photoreceptors is now consistent with the reduction in the expression level of these genes. The results are well correlated with our analysis regarding the altered fate specification of retinal cells in the Pax6^−/−^ retina.

Previously, the role of Pax6 in axon guidance has been documented in thalamocortical (TCA), corticofugal axons (CFA) and Tract of the Postoptic Commissural (TPOC) axons by regulating r-cadherin and classic axon guidance molecules^8,28^. In line with this, various reports suggest that Pax6 controls neuronal migratory patterns by regulating the expression of specific guidance cues in the developing telencephalon^8,11,29,30^. Additionally, Pax6 also regulates the expression of cell adhesion molecules, where the loss of Pax6 in the ventral telencephalon leads to the misexpression of several cell surface molecules that could restrict the thalamic axons from growing through the internal capsule^8^. In retina, it is reported that over-expression of Pax6 could lead to aberrant axonal patterns where the axons fail to navigate towards the optic chiasm, but the exact mechanism by which this happens is not known^9^. However, the role of Pax6 in the regulation of intra-retinal axon guidance and how it is mediating the function is mostly unexplored.

Here, we demonstrate that Pax6 could regulate the ECM molecules, especially the collagen subunits, which serve as a basal membrane on which the retinal axons grow, fasciculate and extend towards the optic disc region. In this scenario, we have observed a severe up-regulation of ECM molecules in Pax6^−/−^ retina. Other possibilities for the misguidance of the axons can be the difficulty in accessing diffused inhibitory molecules secreted by retinal cells because of a thicker ECM and hence the axons might fail to respond to those cues. Moreover, we have not observed any increase in the number of astrocytes in the Pax6^−/−^ retina ruling out the possibility of glial cell accumulation, but we do not rule out the possibility of abnormal activation of glial cells for the deposition of ECM. Matrix proteases could also play a significant role in the altered axonal outgrowth because they act on ECM molecules to reveal cues for the proper neurite guidance by exposing axons to the secretory or immobilized axon guidance molecules. Even the classic axon guidance molecules such as ephrins, semaphorins and netrins could influence either promotion or inhibition of the outgrowth of axons through the interaction of integrins with the cell adhesion molecules^31^. Up-regulation of collagen subunits could also anchor the proteoglycans like HSPG and glycosaminoglycans like chondroitin sulphates leading to abnormal patterning of neuronal projections. It is also known that HSPGs are essential to exert chemorepulsion towards the growing axons through Slit signaling^32^. This could generate an unfavorable condition for the axonal outgrowth and ultimately inhibit the axon growth and fasciculation^33^. Abnormal deposition of few collagen molecules such as collagen I, III and IV was reported in Cynomolgus monkey, which has caused optic nerve damage^34^. Other reports have also stated that increased collagen deposition could lead to an age-related axonal loss in human subjects^35^. Axons of the DRG neurons are typically known to grow in the presence of laminin, Col1, Col4 but Col5a2 which is a fibril-forming collagen blocks the axonal outgrowth explaining its inhibitory effect^36^.

In addition to ECM molecules, we have also shown that Pax6 directly regulates EphB1 and Sema5b. Up-regulation of EphB1 and down-regulation of Sema5B in Pax6^−/−^ retina could have implications in the disparities observed in axon-axon interaction. Up-regulation of EphB1 receptors on the RGC axons might also affect the interaction between the axons so that the efficiency of fasciculation decreases. In the brain, EphB forward signaling is required for the proper navigation of axon growth cones^37^. Whereas in the retina, this signaling can act as both reverse and forward signaling at the optic cup and at the visual target, superior colliculus, respectively^38^. Previously it is demonstrated that EphB1 is expressed by a subset of RGCs that are present exclusively in the ventrotemporal (VT) retinal axons that project ipsilaterally to establish the binocular vision. Up-regulation of EphB1 in Pax6^−/−^ retina could attribute to either overexpression of EphB1 receptors on the VT retinal axons or its atypical expression on the dorsotemporal and nasal RGC axons. This could promote the axons to be guided ipsilaterally rather than contralaterally at the optic chiasm region, which is beyond the scope of this study^38,39^. Severe reduction in the expression of Sema5B observed in Pax6^−/−^ retina might be due to the loss of cells that express Sema5B in the Onbl and also, we could not delineate its function or cell types that express it since establishing proper retinal lamination is an early postnatal function^40^. Our results indicate that there is definite alteration in the expression of a group of axon guidance genes that are directly regulated by Pax6 which could also be the reason for the observed altered axon guidance.

In summary, we conclude that Pax6 expression post fate specification of RGCs is necessary for regulating the expression of axon guidance genes and most importantly for maintaining a conducive ECM through which the nascent axons get guided and fasciculate to reach the optic disc.

## Methods

### Generation of plasmid vectors

Mouse Sema5b promoter (~ 2.5 kb) was PCR amplified from mouse genomic DNA using specific primers (Table S4) and the PCR product was cloned into a pCRII TA cloning vector to generate pCRII_Sema5b vector. Further, the pSema5b_luc vector was constructed by digesting pCRII_Sema5b with *KpnI/XhoI* and cloned into the minimal promoter PGL2_luc vector digested with *KpnI/XhoI* . The pEphb1_luc construct was a kind gift from Prof. Oscar Marin, King’s College London. PGV Basic vector is a promoter less luciferase vector procured from Promega. pCAGIG-Pax6^41^ was used to over-express Pax6, and Pax6 was down-regulated using different siRNA oligos cloned into the iLenti-siRNA-GFP vector using specifically designed oligos (Table S4) according to manufacturer’s protocol (Applied Biological Materials, Inc). siRNA oligo insertion into iLenti-siRNA-GFP vector was further confirmed by sequencing using ABI sequencing protocol.

### Animal models and breeding

Mice were maintained on either Swiss or FVB or C57Bl/6 genetic background or mixed. Noon of the day on which the vaginal plug was found was designated as E0.5. All animal procedures including breeding and Knockout generation were performed according to the protocols approved by the Institutional Animal Ethics Committee (IAEC) of Rajiv Gandhi Centre for Biotechnology (IAEC/514/JAC/2016 and IAEC/647/JAC/2017) and were cared for as per guidelines. They were maintained in a 12 h light/dark cycle and provided normal mouse diet *ad labitum* throughout this study. Mouse lines used in our study were *ROSA26-CreER*^*T2*^* (B6.129-Gt(ROSA)26Sor*^*tm1 (cre/ERT2)Tyj*^/J, JAX# 008463) *and Pax6*^*fl/fl*^ mice. The *ROSA26-CreER*^*T2*^ animal was procured from the National Center for Biological Sciences (NCBS) Bangalore animal facility, which expresses Cre Recombinase only upon the administration of Tamoxifen in every cell. *Pax6*^*fl/fl*^ mouse line was procured from Dr. Shubha Tole’s lab, TIFR, Mumbai which was initially generated and provided by Prof. David J Price (Edinburg University). In these mice, exon 5 and 6 of the Pax6 locus was flanked by loxP sites, this region codes for the Paired domain of the Pax6 protein, located at the N-terminal side of the protein. When the Cre recombinase is active, it results in the looping out of Exon 5 and 6, which does not create any frameshift mutation in the coding region, whereby the protein is having an intact C-terminal but is truncated. By crossing the *Pax6*^*fl/fl*^ mice with the *ROSA26-CreER*^*T2*^ line, we have generated a new transgenic *ROSA26-CreER*^*T2*+*/−*^*;Pax6*^*fl/fl*^line, which was in turn crossed with *Pax6*^*fl/fl*^ females to generate embryos carrying double *Pax6* floxed alleles. Tamoxifen administration generated a conditional knock-out mice *(*deletion of Pax6; named *ROSA26-CreER*^*T2*^*;Pax6*^−/−)^, carrying a recombinant Pax6 locus in the Cre expressing cells and lacking functional Pax6 in the retina. The animals which are negative for Cre expression from the littermates are considered as controls. All embryos taken for this study were devoid of pigmentation in their eyes indicating an albino trait. This was important since the *ROSA26-CreER*^*T2*+*/−*^*;Pax6*^*fl/fl*^ line was derived from a mixed background. *Pax6*^−/−^ embryos die *perinatally* and the dead pups were excluded thereby, limiting the ages that could be analyzed.

### Genotyping and tamoxifen administration

Genotyping of mice were performed by PCR with the Cre specific primers and primers specific for the presence of loxP region in the Pax6^*fl/fl*^ animal to identify homozygous Pax6^*fl/fl*^ animals (Table S4). Wild type, heterozygous and homozygous embryos for Pax6^*fl/fl*^ animals were confirmed with the genotyping PCR yielding a band size of 280 bp, 280 bp & 350 bp and 350 bp respectively. Single dose of 8–10 mg of tamoxifen (Sigma-Aldrich) in corn oil was administered by intraperitoneal injection to pregnant females (*Pax6*^*fl/fl*^ females bred with *ROSA26-CreER*^*T2*+*/−*^*;Pax6*^*fl/fl*^ males) at E15.5 (around noon) and the animals were sacrificed by cervical dislocation at E18.5 and the embryos were collected.

### Immunohistochemistry

Immunohistochemical analysis was carried out on the eye sections as described earlier^42^ using the antibodies listed in Table S4. Briefly, 4% paraformaldehyde fixed sections were permeabilized using 0.2% (for cytoplasmic antigens) or 0.4% (for nuclear antigens) TritonX-100 in 1X PBS containing 5% NGS, followed by overnight incubation with primary antibodies at 4 °C, washed with 1X PBS and incubated with respective secondary antibody. The nucleus was visualized using DAPI counterstaining. For Pax6 antibody that recognizes the N-terminal (DSHB Cat# Pax6, RRID:AB_528427), antigen retrieval was carried out and it does not recognize the truncated Pax6 protein (Fig. 3H–S). C-terminal recognizing Pax6 antibody (Millipore Cat# AB2237, RRID:AB_1587367) was used to label both normal and truncated Pax6 co-localized with cell specific antibodies (Fig S1C & Fig S2C). After 4 days in culture, the explants were washed with 1X PBS and fixed with 4% PFA for 30 min at 4 ºC and permeabilized for 30 min followed by overnight incubation with mouse anti-SMI-31 antibody. For immunohistochemical analysis of the retinal flatmounts, the eyes were fixed in 4% PFA for 10 min at room temperature and the retina was dissected and mounted on a glass slide. The flatmount was blocked using 0.1% TritonX-100 in 1X PBS containing 5% NGS and immunohistochemical analysis was carried out with rabbit anti-β III tubulin antibody. Images were captured using a confocal microscope. Counting of positive and negative cells was carried out in a blinded manner. Details of antibodies used are provided in Table S4.

### Gene expression analysis

For gene expression analysis, the retinal tissues were dissected out and the total RNA was isolated using TRI-reagent (Sigma) and cDNA synthesis was carried out with ~ 2 μg of total RNA. Relative expression analysis was carried out using SYBR green premix (Takara) and gene-specific primers (Table S4) along with β-actin as the internal control. Real-time analyses were done with 2^-ΔΔct^ method.

### Chromatin Immunoprecipitation (ChIP)

ChIP analysis was performed on E18 retinal tissue with rabbit anti-Pax6 antibody (5–10 µg), and anti-rabbit IgG (5–10 µg) antibody using a modified protocol of Sun et al., 2015^43^. Briefly, E18 retina was dissected out and cross-linked with 1.5% formaldehyde for 8 min at room temperature and the fixed tissues were homogenized and sonicated. Immunoprecipitation was carried out with the sonicated lysate and the final DNA pellet was dissolved in water and used for downstream PCR amplification.

### Ex-vivo electroporation and retinal explant culture

16-day pregnant mice were sacrificed and E16 embryos were dissected out in 1X HBSS. Approximately 0.5–1 μl of Pax6 siRNA expressing plasmid constructs (1 μg/μl) and control plasmids were injected separately into the vitreous chamber of the embryos using a microcapillary tube connected to a micromanipulator. Five Pulses each of 50 ms, 42 V and 950 ms interval were delivered from a BTX, ECM 803 Electro square electroporator using a 3 mm platinum tweezer electrode with the negative electrode placed on the side of the lens of the injected eye so that the cells in the GCL alone gets electroporated. After the electroporation, the eyes were enucleated and retina was dissected out in a sterile condition. The retina was then made into smaller pieces and cultured on a PLL (20 µg/ml)/laminin (10 µg/ml) coated coverslip in the retinal explants culture medium (Neurobasal medium with 0.4% methylcellulose, 0.5% Glutamax, 2% B27 and 0.2% fungizone) for four days to visualize the growing axons. For the co-culture experiment, retinal explants of E18 Pax6^fl/fl^ and Pax6^−/−^ mice were cultured individually along with E18 GFP mice retina on a PLL/laminin coated coverslip in a 24 well culture plate and analyzed at different time intervals for up to 14 days.

### RNA-Seq and data analysis

Retinal tissues from both Pax6^fl/fl^ and Pax6^−/−^ mice were dissected and homogenized with TRI reagent. The total RNA was extracted and treated with DNaseI to eliminate DNA contamination. RNA-seq was performed on an Illumina HiSeq platform. The pre-processed reads were aligned to the *Mus musculus* genome downloaded from Ensembl database (ftp://ftp.ensembl.org/pub/release94/fasta/mus_musculus/dna/Mus_musculus.GRCm38.dna.toplevel.fa.gz). The alignment was performed using Hisat2 program (version 2.0.5) with default parameters. After aligning the reads with the reference gene model, the aligned reads were used for estimating expression of the genes and transcripts, using cufflinks program (version 2.2.1). A volcano plot was used to illustrate the magnitude of fold change of signature genes which are labelled with gene symbol, using ggplot2 package in R. Genes with p < 0.05, Fold change > 1.5 were used to generate heatmap depicting changes in gene expression, using gplots package in R. Gene Set Enrichment analysis (GSEA) was done using log_2_ fold change as ranking metric in pre-ranked mode with ‘classic’ as enrichment score setting^44^. GO analysis by statistical overrepresentation test was performed using the PANTHER gene list analysis tool. Reference gene lists and annotated information were obtained from the Panther database^45^.

### Luciferase analysis

Dual luciferase assay was carried out in Neuro-2a (N2A) cells according to the manufacturer’s protocol (Promega, Cat. no. E1910) using a Luminometer (TD20/20, Promega). Authenticated N-2a cell line was obtained from National Centre for Cell Science (NCCS) cell repository, Pune, India.. Briefly, N2A cells (P4) cultured in DMEM containing 10% FBS, 1% Glutamax, 1% NEAA and 1% sodium pyruvate, were transfected with the pEphB1_luc construct, pCAGIG-Pax6, Pax6 siRNA constructs along with pRL-Tk vector in a 24-well plate using Lipofectamine 2000. PGV Basic vector (Promega) is used as a control and Renilla luciferase was used for normalization. All the values are expressed as the mean ± standard deviation (SD) of at least three independent experiments carried out in triplicates.

### Quantification and statistical analysis

We followed a common practice to check the normality distribution of datasets using Shapiro–Wilk’s test. If data is expected to follow the normal distribution, we performed student’ t-test. For data not supporting normal distribution, we performed Wilcoxon test. (*p < 0.05; **p < 0.01; ***p < 0.001). R program and Microsoft Excel have been used to perform the aforementioned statistical tests.

## Supplementary information

Supplementary Legends

Supplementary file2

## Data Availability

We agree to make aviliable materials, data and associated protocols used in this study upon request.

## References

[1] Young RW (1985). Cell differentiation in the retina of the mouse. Anat. Rec..

[2] Bao ZZ (2008). Intraretinal projection of retinal ganglion cell axons as a model system for studying axon navigation. Brain Res..

[3] Erskine L, Herrera E (2014). Connecting the retina to the brain. ASN Neuro.

[4] Jagatha B (2009). In vitro differentiation of retinal ganglion-like cells from embryonic stem cell derived neural progenitors. Biochem. Biophys. Res. Commun..

[5] Divya MS (2017). Intraocular injection of ES cell-derived neural progenitors improve visual function in retinal ganglion cell-depleted mouse models. Front Cell Neurosci..

[6] Simpson TI, Price DJ (2002). Pax6; a pleiotropic player in development. BioEssays.

[7] Stoykova A, Gruss P (1994). Roles of Pax-genes in developing and adult brain as suggested by expression patterns. J. Neurosci..

[8] Jones L, Lopez-Bendito G, Gruss P, Stoykova A, Molnar Z (2002). Pax6 is required for the normal development of the forebrain axonal connections. Development.

[9] Manuel M, Pratt T, Liu M, Jeffery G, Price DJ (2008). Overexpression of Pax6 results in microphthalmia, retinal dysplasia and defective retinal ganglion cell axon guidance. BMC Dev. Biol..

[10] Sebastian-Serrano A (2012). Palphax6 expression in postmitotic neurons mediates the growth of axons in response to SFRP1. PLoS ONE.

[11] Simpson TI, Pratt T, Mason JO, Price DJ (2009). Normal ventral telencephalic expression of Pax6 is required for normal development of thalamocortical axons in embryonic mice. Neural Dev..

[12] Hubert T, Grimal S, Carroll P, Fichard-Carroll A (2009). Collagens in the developing and diseased nervous system. Cell Mol. Life Sci..

[13] Myers JP, Santiago-Medina M, Gomez TM (2011). Regulation of axonal outgrowth and pathfinding by integrin-ECM interactions. Dev. Neurobiol..

[14] Marquardt T (2001). Pax6 is required for the multipotent state of retinal progenitor cells. Cell.

[15] Drager UC (1985). Birth dates of retinal ganglion cells giving rise to the crossed and uncrossed optic projections in the mouse. Proc. R. Soc. Lond. B Biol. Sci..

[16] Bumsted O'Brien KM (2004). Expression of photoreceptor-specific nuclear receptor NR2E3 in rod photoreceptors of fetal human retina. Invest. Ophthalmol. Vis. Sci..

[17] de Gooyer TE (2006). Rod photoreceptor loss in Rho^−^^/^^−^ mice reduces retinal hypoxia and hypoxia-regulated gene expression. Invest. Ophthalmol. Vis. Sci..

[18] Feng L (2006). Requirement for Bhlhb5 in the specification of amacrine and cone bipolar subtypes in mouse retina. Development.

[19] Liu Q (2012). Expression of wild-type Rp1 protein in Rp1 knock-in mice rescues the retinal degeneration phenotype. PLoS ONE.

[20] Ochocinska MJ (2012). NeuroD1 is required for survival of photoreceptors but not pinealocytes: results from targeted gene deletion studies. J. Neurochem..

[21] Sahaboglu A (2017). Temporal progression of PARP activity in the Prph2 mutant rd2 mouse: Neuroprotective effects of the PARP inhibitor PJ34. PLoS ONE.

[22] Muto A, Iida A, Satoh S, Watanabe S (2009). The group E Sox genes Sox8 and Sox9 are regulated by Notch signaling and are required for Muller glial cell development in mouse retina. Exp Eye Res..

[23] Honda A (2017). Extracellular signals induce glycoprotein m6a clustering of lipid rafts and associated signaling molecules. J Neurosci.

[24] Madsen CD (2015). Hypoxia and loss of PHD2 inactivate stromal fibroblasts to decrease tumour stiffness and metastasis. EMBO Rep..

[25] Schneidman-Duhovny D, Inbar Y, Nussinov R, Wolfson HJ (2005). Geometry-based flexible and symmetric protein docking. Proteins.

[26] Philips GT (2005). Precocious retinal neurons: Pax6 controls timing of differentiation and determination of cell type. Dev. Biol..

[27] Hatakeyama J, Tomita K, Inoue T, Kageyama R (2001). Roles of homeobox and bHLH genes in specification of a retinal cell type. Development.

[28] Andrews GL, Mastick GS (2003). R-cadherin is a Pax6-regulated, growth-promoting cue for pioneer axons. J. Neurosci..

[29] Mastick GS, Davis NM, Andrew GL, Easter SS (1997). Pax-6 functions in boundary formation and axon guidance in the embryonic mouse forebrain. Development.

[30] Pratt T (2000). A role for Pax6 in the normal development of dorsal thalamus and its cortical connections. Development.

[31] Nakamoto T, Kain KH, Ginsberg MH (2004). Neurobiology: new connections between integrins and axon guidance. Curr. Biol..

[32] Hu H (2001). Cell-surface heparan sulfate is involved in the repulsive guidance activities of Slit2 protein. Nat. Neurosci..

[33] Xiao T, Baier H (2007). Lamina-specific axonal projections in the zebrafish tectum require the type IV collagen Dragnet. Nat. Neurosci..

[34] Morrison JC, L'Hernault NL, Jerdan JA, Quigley HA (1989). Ultrastructural location of extracellular matrix components in the optic nerve head. Arch. Ophthalmol..

[35] Hernandez MR, Luo XX, Andrzejewska W, Neufeld AH (1989). Age-related changes in the extracellular matrix of the human optic nerve head. Am. J. Ophthalmol..

[36] Chernousov MA, Stahl RC, Carey DJ (2001). Schwann cell type V collagen inhibits axonal outgrowth and promotes Schwann cell migration via distinct adhesive activities of the collagen and noncollagen domains. J. Neurosci..

[37] Egea J, Klein R (2007). Bidirectional Eph-ephrin signaling during axon guidance. Trends Cell Biol..

[38] Williams SE (2003). Ephrin-B2 and EphB1 mediate retinal axon divergence at the optic chiasm. Neuron.

[39] Petros TJ, Shrestha BR, Mason C (2009). Specificity and sufficiency of EphB1 in driving the ipsilateral retinal projection. J. Neurosci..

[40] Matsuoka RL (2011). Class 5 transmembrane semaphorins control selective Mammalian retinal lamination and function. Neuron.

[41] Divya TS (2016). Regulation of Tlx3 by Pax6 is required for the restricted expression of Chrnalpha3 in Cerebellar Granule Neuron progenitors during development. Sci Rep..

[42] Rasheed VA (2014). Developmental wave of Brn3b expression leading to RGC fate specification is synergistically maintained by miR-23a and miR-374. Dev. Neurobiol..

[43] Sun J (2015). Identification of in vivo DNA-binding mechanisms of Pax6 and reconstruction of Pax6-dependent gene regulatory networks during forebrain and lens development. Nucleic Acids Res..

[44] Subramanian A (2005). Gene set enrichment analysis: a knowledge-based approach for interpreting genome-wide expression profiles. Proc Natl Acad Sci USA.

[45] Mi H, Muruganujan A, Ebert D, Huang X, Thomas PD (2019). PANTHER version 14: more genomes, a new PANTHER GO-slim and improvements in enrichment analysis tools. Nucleic Acids Res..

