# Supplementary material for: Pax6 modulates intra-retinal axon guidance and fasciculation of retinal ganglion cells during retinogenesis
Source: Sci Rep. 2020 Sep 30;10:16075. doi: 10.1038/s41598-020-72828-4 (PMC7527980; doi:10.1038/s41598-020-72828-4)
Supplement: Supplementary file 1 — Supplementary Legends [file 41598_2020_72828_MOESM1_ESM.docx]

**Supplementary figures:**

**Fig. S1: Analysis of different cell fates in the Pax6^-/-^** **retina**

Immunohistochemical analysis with AP2α, recoverin and calbindin showed a significant reduction in the number of amacrine cells (B and F), photoreceptors (J and L) and horizontal cells (N and P) in Pax6^-/-^ retina compared Pax6^fl/fl^ (Q). n=3, 3 embryos of each control and cKO were taken and 3 sections of each were counted. Scale bar - 25µm. Error bars indicate SEM from three biological replicates (p<0.05).

**Fig. S2: RGC fate specification in Pax6^-/-^** **retina**

Expression of the RGC marker Brn3 showed a drastic reduction in the number of RGCs (B and F) in Pax6^-/-^ retina compared Pax6^fl/fl^ (I). n=3, 3 embryos of each control and cKO were taken and 3 sections of each were counted. Scale bar - 25µm. Error bars indicate SEM from three biological replicates (p<0.05).

**Fig. S3: Prediction of collagen complex structures**

# (A-J): Predicted structures of collagen complexes with symmetric multimolecular assemblies C(N) by geometry-based docking using PDB 5CTD_B chain. (J): Atomic Contact Energies (ACE) score of predicted structures of multimeric complexes of Col1a2 (PDB:5CTD, B chain) by symmetrical protein-protein docking.

# Table S4: List of primers and antibodies used in the study
