# Supplementary material for: Pax6 modulates intra-retinal axon guidance and fasciculation of retinal ganglion cells during retinogenesis
Source: Sci Rep. 2020 Sep 30;10:16075. doi: 10.1038/s41598-020-72828-4 (PMC7527980; doi:10.1038/s41598-020-72828-4)
Supplement: Supplementary file 2 — Supplementary file2 [file 41598_2020_72828_MOESM2_ESM.pdf]

Pax6 regulates intra-retinal axon guidance and fasciculation of retinal ganglion cells during retinogenesis

Soundararajan Lalitha[1,2], *Budhaditya Basu*[1], *Suresh Surya*[1], *Vadakkath Meera*[1,2], *Paul Ann Riya*[1,2], *Surendran Parvathy* [1,2], *Ani Venmanad Das*[3], *Krishnankutty Chandrika Sivakumar*[4], *Shijulal Nelson-Sathi*[5] *and Jackson James*[1,2*]

[1] Neuro Stem Cell Biology Laboratory, Neurobiology Division, Rajiv Gandhi Centre for Biotechnology, Thiruvananthapuram, Kerala-695 014, India

[2] Research Centre, The University of Kerala, Thiruvananthapuram, Kerala-695 014, India

[3] Cancer Biology Programs-12, Rajiv Gandhi Centre for Biotechnology, Thiruvananthapuram, Kerala-695 014, India

[4] Bioinformatics Facility, Rajiv Gandhi Centre for Biotechnology, Thiruvananthapuram, Kerala-695 014, India

[5] Interdisciplinary Biology, Rajiv Gandhi Centre for Biotechnology, Thiruvananthapuram, Kerala-695 014, India

Figure-S1

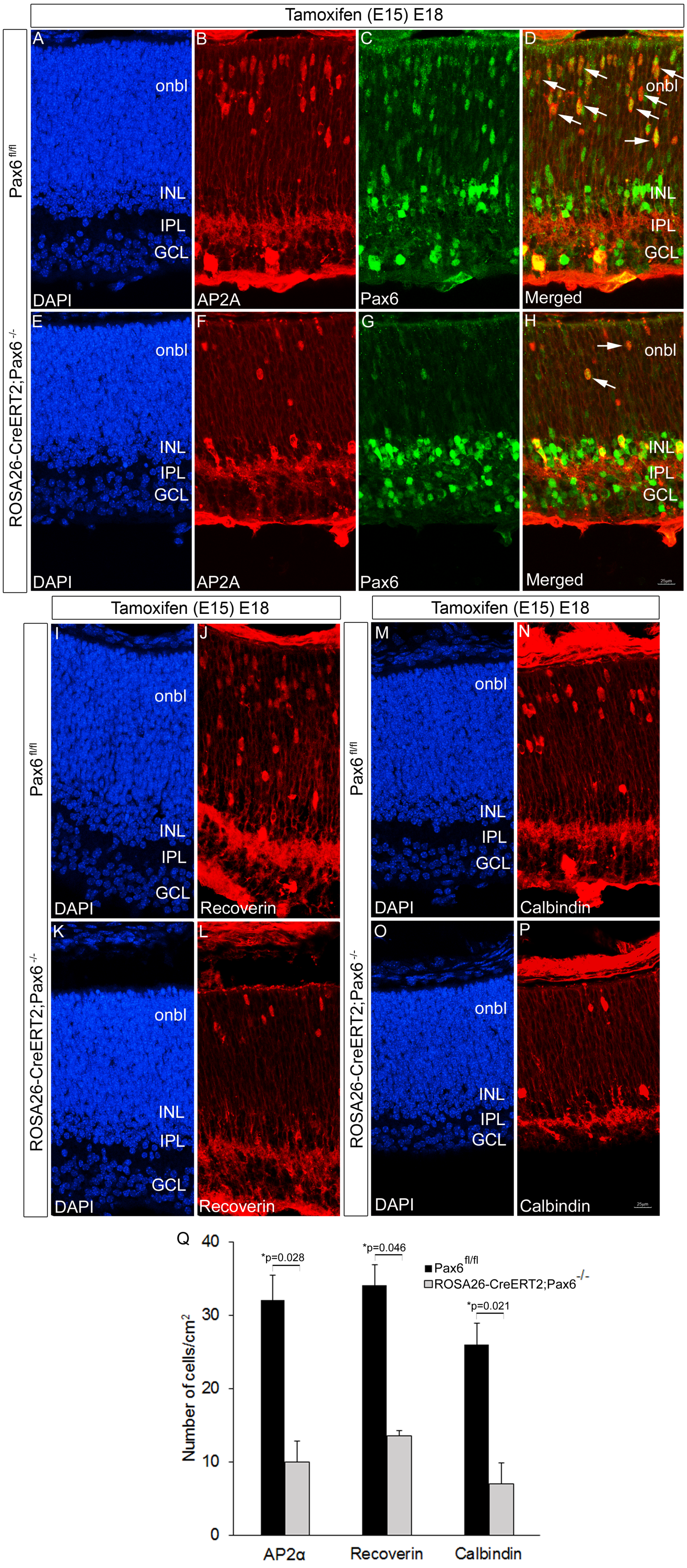

Tamoxifen (E15) E18
Pax6 fl/fl
ROSA26-CreERT2;Pax6 -/-
A
B
C
D
onbl
INL
IPL
GCL
DAPI
AP2A
Pax6
Merged
E
F
G
H
Tamoxifen (E15) E18
Tamoxifen (E15) E18
I
J
K
L
M
N
O
P
Recoverin
Calbindin
Q
Number of cells/cm2
*p=0.028
*p=0.046
*p=0.021
Pax6 fl/fl
ROSA26-CreERT2;Pax6 -/-
40
30
20
10
0
AP2α
Recoverin
Calbindin

Figure-S2

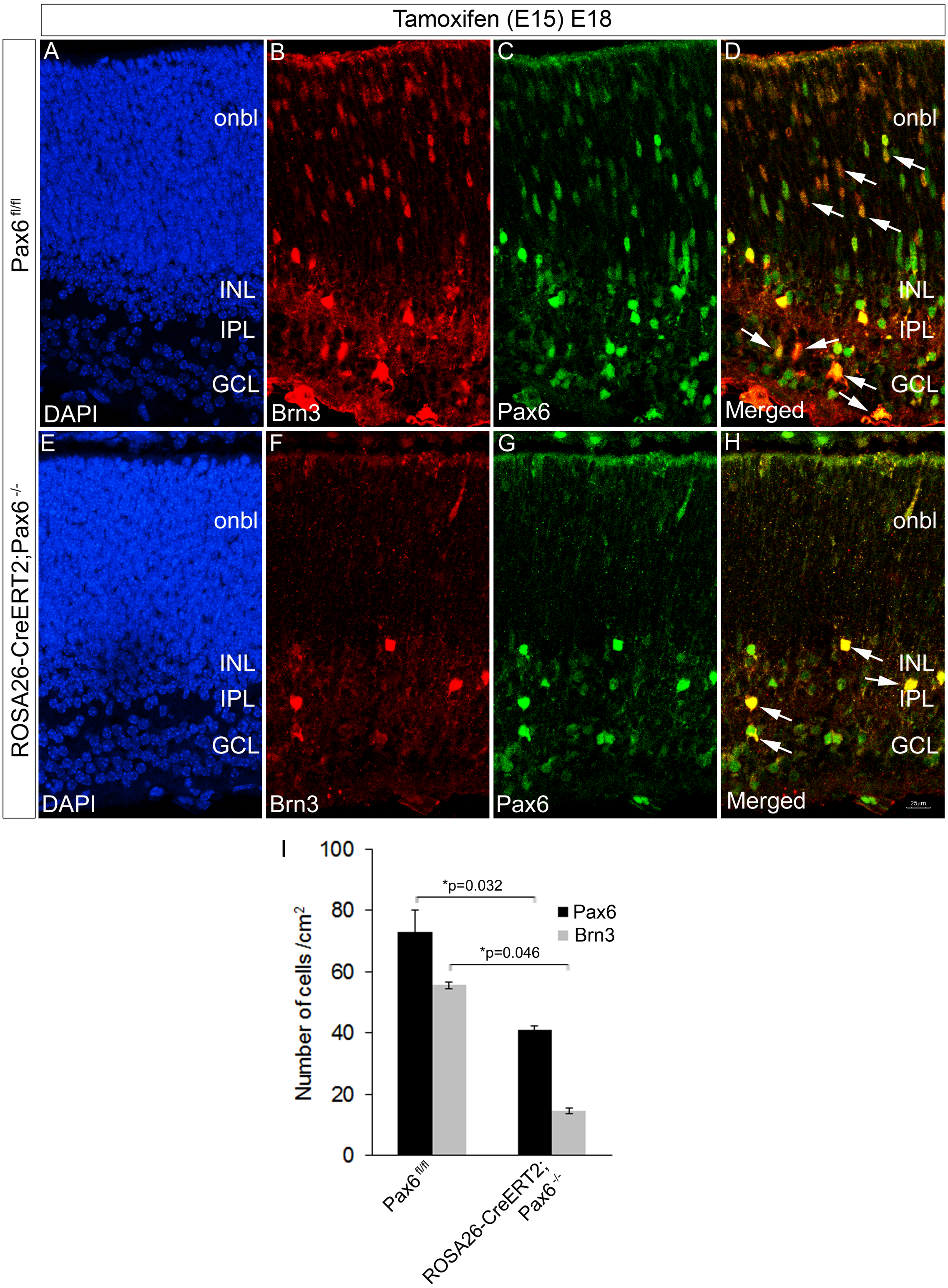
Tamoxifen (E15) E18
Pax6 fl/fl
ROSA26-CreERT2;Pax6 -/-
A
B
C
D
E
F
G
H
onbl
INL
IPL
GCL
DAPI
Brn3
Pax6
Merged
25μm
I
100
80
60
40
20
0
Number of cells /cm2
*p=0.032
*p=0.046
Pax6
Brn3
Pax6 fl/fl
ROSA26-CreERT2; Pax6 -/-

Figure-S3

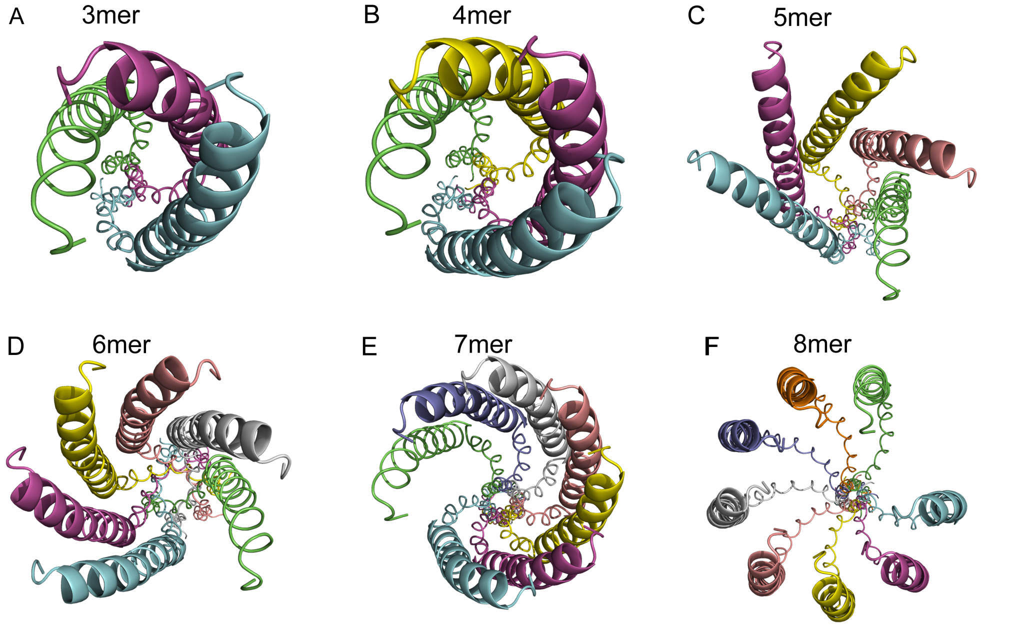

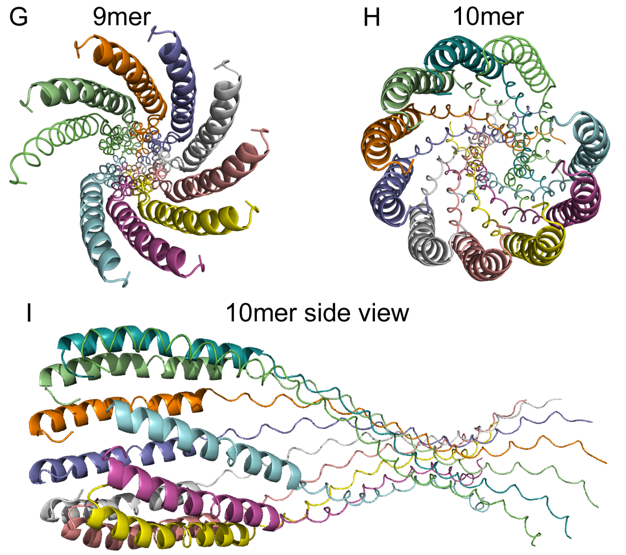

J

Symmetric order	ACE
3	15.14
4	-380.45
5	-389.35
6	-245.82
7	-121.40
8	-1117.28
9	-832.59
10	-1267.83

Table S4

List of primers used in the study:

Primer	Sequence	Product size
Pax6 siRNA1 F	AAAAGCAGACGGCATGTATGATAAACTAAGGAT	
Pax6 siRNA1 R	AAAGATCCTTAGTTTATCATACATGCCGTCTGC	
Pax6 siRNA2 F	AAAAACTCCTAGTCACATTCCTATCAGCAGCAG	
Pax6 siRNA2 R	AAAGCTGCTGCTGATAGGAATGTGACTAGGAGT	
Pax6 siRNA3 F	AAAAGCCCAGCTTCACCATGGCAAACAACCTGC	
Pax6 siRNA3 R	AAAGGCAGGTTGTTTGCCATGGTGAAGCTGGGC	
Pax6 siRNA4 F	AAAAGGAGTGAACCTGACATGTCTCAGTACTGG	
Pax6 siRNA4 R	AAAGCCAGTACTGAGACATGTCAGGTTCACTCC	
Sema5b Promoter F	AAAGAATCTGGGGAGGGAAGGTGTG	2.5kb
Sema5b Promoter R	AGAGGCTGGACACTAAGAAAGGGG	
pPax6 ChIP F	AGAGCCGAAAACAAGTGTATTC	269bp
pPax6 ChIP R	ATTAGCGAAGCCTGACCTC	
pSema5B ChIP F	GTTCCAGGTCATGCAGTTC	274bp
pSema5B ChIP R	CTAACCGGGAATGAAAGATGG	
pEphB1 ChIP F	GCAGTTCTCCTAAATCACCAC	284bp
pEphB1 ChIP R	GTCACATTGCGAGCTACAG	
Pax6 fl/fl F	AAATGGGGGTGAAGTGTGAG	280 & 350bp
Pax6 fl/fl R	GGAGCAGTCCTTCACCTCTG	
Cre F	CGATGCAACGAGTGATGAGGTTC	345bp
Cre R	GCACGTTCACCGGCATCAAC	
Ephb1 F	ACGGAAGTCAGGAGCTTTG	303bp
Ephb1 R	CTCATAGCCAGGCTTACAGG	
Sema5b F	TGAGATTCCTGTCATCCTACC	310bp
Sema5b R	GTAGAAGTTGGCTCTGTCATC	
Pax6 F	CCAACGGTTGTGTGAGTAAAATTC	258bp
Pax6 R	GCTTTTCGCTAGCCAGGTTGCGAAGAAC	
Beta actin F	AGACTTCGAGCAGGAGATG	322bp
Beta actin R	CTTGATCTTCATGGTGCTAGG	

List of antibodies used in the study:

Antibody	Dilution	Source	Identifier
Rabbit anti-Pax6	1:200	Millipore	(Millipore Cat# AB2237, RRID:AB_1587367)
Mouse anti-Pax6	1:200	DSHB	(DSHB Cat# pax6, RRID:AB_528427)
Goat anti-Brn3	1:100	Santa Cruz	(Santa Cruz

			Biotechnology Cat# sc-6026, RRID:AB_673441)
Mouse anti-Ap2α	1:50	DSHB	(DSHB Cat# 5E4, RRID:AB_2056333)
Mouse anti-Recoverin	1:500	Abcam	(Abcam Cat# ab31928, RRID:AB_882309)
Mouse anti-Calbindin	1:1000	Sigma	(Sigma-Aldrich Cat# C9848, RRID:AB_476894)
Mouse anti-SMI31	1:1000	Covance	(Covance Research Products Inc Cat# SMI-31R-100, RRID:AB_10122491)
Rabbit anti-β III tubulin	1:500	Millipore	AB5564
Goat anti-Rabbit IgG (H+L) Cross-Adsorbed Secondary Antibody, Alexa Fluor 488	1:200	Invitrogen	(Molecular Probes Cat# A-11008, RRID:AB_143165)
Cy™3 AffiniPure F(ab')$_2$ Fragment Goat Anti-Rabbit IgG (H+L)	1:400	Jackson Immunoresearch	(Jackson ImmunoResearch Labs Cat# 111-166-003, RRID:AB_2338007)
Cy™3 AffiniPure F(ab')$_2$ Fragment Goat Anti-Mouse IgG (H+L)	1:400	Jackson Immunoresearch	(Jackson ImmunoResearch Labs Cat# 115-166-003, RRID:AB_2338699)
Cy™3 AffiniPure F(ab')$_2$ Fragment Donkey Anti-Goat IgG (H+L)	1:400	Jackson Immunoresearch	(Jackson ImmunoResearch Labs Cat# 705-165-003, RRID:AB_2340411)
